# Acute liver dysfunction after cardiac arrest

**DOI:** 10.1371/journal.pone.0206655

**Published:** 2018-11-05

**Authors:** Enrica Iesu, Federico Franchi, Federica Zama Cavicchi, Selene Pozzebon, Vito Fontana, Manuel Mendoza, Leda Nobile, Sabino Scolletta, Jean-Louis Vincent, Jacques Creteur, Fabio Silvio Taccone

**Affiliations:** 1 Department of Intensive Care, Erasme Hospital, Université Libre de Bruxelles, Brussels, Belgium; 2 Department of Medical Biotechnologies, Anesthesia and Intensive Care Unit, University of Siena, Siena, Italy; 3 Department of Intensive Care, Hospital Universitari de Tarragona Joan XXIII, Tarragona, Spain; University of Tübingen, GERMANY

## Abstract

Few data are available regarding hypoxic hepatitis (HH) and acute liver failure (ALF) in patients resuscitated from cardiac arrest (CA). The aim of this study was to describe the occurrence of these complications and their association with outcome. All adult patients admitted to the Department of Intensive Care following CA were considered for inclusion in this retrospective study. Exclusion criteria were early death (<24 hours) or missing biological data. We retrieved data concerning CA characteristics and markers of liver function. ALF was defined as a bilirubin >1.2 mg/dL and an international normalized ratio ≥1.5. HH was defined as an aminotransferase level >1000 IU/L. Neurological outcome was assessed at 3 months and an unfavourable neurological outcome was defined as a Cerebral Performance Categories (CPC) score of 3–5. A total of 374 patients (age 62 [52–74] years; 242 male) were included. ALF developed in 208 patients (56%) and HH in 27 (7%); 24 patients developed both conditions. Patients with HH had higher mortality (89% vs. 51% vs. 45%, respectively) and greater rates of unfavourable neurological outcome (93% vs. 60% vs. 59%, respectively) compared to those with ALF without HH (n = 184) and those without ALF or HH (n = 163; p = 0.03). Unwitnessed arrest, non-shockable initial rhythm, lack of bystander cardiopulmonary resuscitation, high adrenaline doses and the development of acute kidney injury were independent predictors of unfavourable neurological outcome; HH (OR: 16.276 [95% CIs: 2.625–81.345; p = 0.003), but not ALF, was also a significant risk-factor for unfavourable outcome. Although ALF occurs frequently after CA, HH is a rare complication. Only HH is significantly associated with poor neurological outcome in this setting.

## Introduction

The outcome of patients after sudden cardiac arrest (CA) remains poor, in particular because a large proportion of these patients will eventually die after hospital admission because of extensive brain damage and cardiogenic shock [1–3]. Moreover, the ischemia-reperfusion injury that occurs after the return of spontaneous circulation (ROSC) contributes to a systemic inflammatory response that has several similarities with sepsis [4] and may result in the development of multi-organ failure (MOF) [5], which further increases the likelihood of poor outcome.

Several studies have suggested that severe cardiovascular and respiratory failure may negatively impact on patient outcome after CA [5,6]. Nevertheless, there is a paucity of data on the prognostic value of extra-cerebral organ dysfunction after CA. In a recent meta-analysis, Sandroni et al. showed that post-arrest acute kidney injury (AKI) had an early onset, occurred in more than 50% of CA patients and was significantly associated with increased mortality [7]. However, there are almost no data on liver dysfunction in this setting; in one recent study, Champigneulle et al. reported that hypoxic hepatitis (HH) occurred in 11% of patients resuscitated after an out-of-hospital CA (OHCA) and, in multivariate analysis, was significantly associated with ICU mortality [8]. Hypoxic liver injury typically occurs in individuals with congestive heart failure and/or low cardiac output but is not always associated with altered liver function [9]. No studies have described the occurrence of acute liver failure (ALF), defined by severely altered coagulation and an increase in total bilirubin, in CA patients.

The aim of this study was, therefore, to describe the occurrence of ALF and HH among CA patients admitted to the intensive care unit (ICU) as well as their association with patient outcome.

## Methods

### Study population

This retrospective study was performed in the Department of Intensive Care at Erasme Hospital, Brussels (Belgium). The local Ethical Committee (Comité d’Ethique Hospitalo-Facultaire Erasme-ULB) approved the study (P2017/264), but waived the need for informed consent because of its retrospective nature. All comatose patients (Glasgow Coma Scale, GCS < 9) admitted after in-hospital CA (IHCA) or OHCA were included in a prospective institutional database (January 2007 to December 2015) and considered as eligible for the study. Exclusion criteria were missing data on liver function or death less than 24 hours after ICU admission, because the occurrence of liver dysfunction could not be fully evaluated in these patients.

### Post-resuscitation care

Our standardized institutional protocol of post-resuscitation management has been extensively described elsewhere [10]. Briefly, all comatose CA patients were treated with targeted temperature management (TTM; target body temperature: 32–34°C) for 24 hours. Deep sedation was obtained using midazolam and morphine, and cisatracurium was administered to control shivering. Rewarming (<0.5°C/h) was achieved passively. Advanced hemodynamic monitoring was used (PiCCO, Pulsion, Munich, Germany) and cardiac function assessed by repeated trans-oesophageal and/or transthoracic echocardiography. Mean arterial pressure was maintained at >65–70 mmHg using volume resuscitation, dobutamine and/or noradrenaline, whenever needed. Intra-aortic balloon counterpulsation (IABP) or extracorporeal membrane oxygenation (ECMO) was also used in cases of severe cardiogenic shock. Ventilation was set to maintain normocapnia and SpO_2_ >94%. Blood glucose was kept between 110 and 150 mg/dl using a continuous insulin infusion. Enteral nutrition was initiated during TTM and continued thereafter according to gastric tolerance. Neurological monitoring and prognostication of coma after CA were performed using a multimodal approach, as previously described [11].

### Data collection

We collected data on demographics (including chronic use of any anticoagulant), pre-existing chronic diseases and cardiopulmonary resuscitation (CPR) (initial rhythm, bystander CPR, time to ROSC, total adrenaline dose) in all patients. Severity of disease was assessed using the Acute Physiology and Chronic Health Evaluation (APACHE) II score [12] and the Sequential Organ Failure Assessment (SOFA) score [13] on the day of admission. To assess liver function, we collected the results from blood samples for aspartate (AST) and alanine (ALT) transaminases, lactate dehydrogenase (LDH, normal values < 200 IU/L), prothrombin time (PT, normal values > 70%), international normalized ratio (INR, normal values ≤ 1.2), total bilirubin (normal values ≤ 1.2 mg/dl) and fibrinogen (normal values > 150 mg/dl) on admission and then once daily until ICU discharge. Use of mechanical ventilation, continuous renal replacement therapy (CRRT) and vasoactive drugs was recorded, as was length of ICU stay. The development of infection during the ICU stay was noted.

Neurological evaluation was assessed at 3 months after CA using the cerebral performance categories score (CPC; 1  =  no or mild neurological disability, 2  =  moderate neurological disability, 3  =  severe neurological impairment, 4  =  vegetative state, 5  =  death) [13]. The CPC evaluation was performed prospectively during follow-up visits or by telephone interview with the general practitioner. Favourable neurological outcome was considered as a CPC 1–2; unfavourable neurological outcome as CPC 3–5 [14].

### Definitions

The definition for ALF was based on previously published criteria, which included an INR ≥1.5, an elevated total bilirubin in the absence of chronic liver disease, and new onset encephalopathy (defined as a patient not obeying orders) [15]. In the presence of acute post-anoxic encephalopathy, we only considered the first two criteria for the definition of ALF.

Hypoxic hepatitis was defined as an increase in AST and/or ALT to more than 20 times the upper normal range (≤ 50 IU/L), i.e., > 1000 IU/L in the setting of acute cardiovascular failure after CA and in the absence of another cause of cell necrosis [16]. We also reported the time to the highest AST and/or ALT values after ICU admission.

AKI was defined according to AKIN criteria [17]. Shock was defined as the need for vasopressor agents for more than 6 hours. We specifically recorded the use of potentially hepatotoxic drugs/interventions (e.g., paracetamol, β-lactams, quinolones, isoniazid, azoles, metrodinazole, any chemotherapy, trimethoprim/ sulfamethoxazole [TMT/SMT] and anti-epileptic drugs).

### Statistical analysis

Discrete variables are expressed as counts (percentage) and continuous variables as median (25th to 75th percentiles). The Kolmogorov-Smirnov test was used, and histograms and normal-quantile plots were examined to verify the normality of distribution of continuous variables. Demographics and clinical differences between groups (HH vs. no-HH; ALF vs. no-ALF; survivors vs. non-survivors; favourable neurological outcome vs. unfavourable outcome) were assessed using the chi-square test, Fisher’s exact test, Student’s t-test, or Mann–Whitney U-test, as appropriate. For the development of HH, and because the number of events was small, only variables with a p values < 0.05 in the univariate analysis were considered in the multivariable logistic regression and odds ratios (ORs) were estimated using a logistic regression approach with penalized profile likelihood based confidence intervals for parameter estimates. For multivariable modelling, correlations between the predictors were checked because high correlations may induce large bias in the estimators of the regression coefficients. Two multivariable logistic regression analyses with ALF or unfavourable neurological outcome as the dependent variables were performed in all patients; co-linearity between variables was excluded prior to modelling; only variables associated with a higher risk of ALF or unfavourable neurological outcome (p <0.2) on a univariate basis were introduced in the multivariable models. ORs with 95% confidence intervals (CI) were computed. A p value < 0.05 was considered as statistically significant. Data were analyzed using IBM SPSS Statistics software, version 22.0 for Windows and R software, version 3.1.0 (CRAN project).

## Results

From a total of 435 patients, 61 were excluded because of early death (n = 51) or the absence of data on liver transaminases, coagulation and/or total bilirubin (n = 10) and 374 patients were analysed (Table 1); 207 (55%) had had an out-of-hospital CA and 221 (59%) a non-shockable initial rhythm. Pre-existing liver disease was present in 17 (4%) patients; 12 of them had alcoholic liver cirrhosis (9 with Child-Pugh B and 3 with Child-Pugh C) and 5 had hepatitis B or C liver cirrhosis (all with Child-Pugh of 3). The ICU length of stay was 4 [2–9] days, 194 (52%) patients died and 227 (61%) patients had an unfavourable neurological outcome.

**Table 1:** Characteristics of the study population, according to the development of hypoxic hepatitis (HH) or acute liver failure (ALF).

	ALL (n = 374)	HH (n = 27)	NO-HH (n = 347)	ALF (n = 208)	NO-ALF (n = 166)
**DEMOGRAPHICS** **Age**, years	62 [52–74]	59 [50–68]	62 [52–75]	61 [51–74]	62 [52–75]
**Weight**, Kg	77 [67–85]	77 [65–85]	77 [68–85]	75 [65–85]	78 [68–88]
**Male**, n (%)	142 (72)	17 (63)	253 (73)	152 (73)	118 (71)
**ICU length of stay**, days	4 [2–9]	3 [2–8]	4 [3–9]	5 [2–10]	4 [2–8]
**APACHE II score**	25 [20–8]	27 [22–9]	24 [20–29]	25 [20–29]	24 [20–28]
**SOFA score**	11 [9–14]	13 [10–15]	11 [9–14]	11 [9–14]	11 [9–13]
**ARREST CHARACTERISTICS**					
**Witnessed**, n (%)	320 (86)	22 (81)	298 (86)	180 (86)	140 (84)
**Bystander CPR**, n (%)	254 (68)	18 (67)	236 (68)	147 (71)	107 (64)
**Time to ROSC**, min	15 [7–25]	21 [15–30]	15 [7–25] **	15 [7–28]	14 [7–21] *
**Adrenaline**, mg	3 [2–5]	4 [3–6]	3 [1–5] *	4 [2–6]	3 [1-] *
**Out-of-hospital**, n (%)	207 (55)	15 (56)	192 (55)	122 (59)	86 (52)
**TTM**, n (%)	331 (89)	23 (85)	308 (89)	196 (94)	135 (81) *
**Non-cardiac cause**, n (%)	153 (41)	13 (48)	140 (40)	93 (45)	60 (36)
**Non-shockable rhythm**, n (%)	221 (59)	21 (78)	200 (58) *	136 (65)	85 (51) **
**ICU mortality**, n (%)	194 (52)	24 (89)	170 (49) **	116 (56)	78 (47)
**Hospital mortality**, n (%)	213 (57)	24 (89)	189 (54) **	128 (61)	85 (51) *
**Unfavourable neurological outcome at 3 months**, n (%)	226 (61)	25 (93)	201 (58) **	133 (64)	93 (56)
**COMORBIDITIES**					
**Chronic heart failure**, n (%)	78 (22)	2 (7)	76 (22) *	54 (26)	24 (14) **
**Hypertension**, n (%)	159 (42)	10 (37)	149 (43)	85 (41)	74 (45)
**Coronary artery disease**, n (%)	146 (39)	7 (26)	139 (40)	83 (40)	63 (38)
**Diabetes,** n (%)	91 (24)	6 (22)	85 (24)	47 (23)	44 (26)
**COPD/asthma**, n (%)	63 (17)	6 (22)	57 (16)	35 (17)	28 (17)
**Neurological disease**, n (%)	54 (14)	2 (7)	52 (15)	25 (12)	29 (18)
**Chronic renal failure**, n (%)	62 (17)	6 (22)	56 (16)	29 (14)	33 (20)
**Liver cirrhosis**, n (%)	17 (4)	1 (4)	16 (5)	14 (7)	3 (2)
**HIV**, n (%)	3 (1)	1 (4)	2 (1)	1 (1)	2 (1)
**Corticosteroids**, n (%) **Chronic anticoagulation, n (%)**	85 (23) 65 (17)	12 (44) 3 (11)	73 (21) ** 62 (18)	55 (26) 40 (19)	30 (18) 25 (15)
**DURING ICU STAY**					
**Infection**, n (%)	241 (64)	18 (67)	223 (64)	137 (66)	104 (63)
**IABP**, n (%)	24 (6)	2 (7)	22 (6)	17 (8)	7 (4)
**ECMO**, n (%)	47 (13)	10 (37)	37 (11) **	37 (18)	10 (6) **
**Shock**, n (%)	200 (53)	25 (93)	175 (50) **	134 (64)	66 (40) **
**Vasopressor therapy**, n (%)	283 (76)	26 (96)	257 (74) **	169 (81)	114 (69) *
**Inotropic agents**, n (%)	201 (54)	25 (93)	176 (51) **	132 (63)	69 (42)
**Mechanical ventilation**, n (%)	374 (100)	27 (100)	347 (100)	206 (99)	163 (98)
** At least one hepatotoxic drug, n (%)**	254 (68)	10 (37)	244 (70) **	142 (68)	112 (67)
**CRRT**, n (%)	61 (16)	14 (52)	47 (14) **	47 (23)	14 (8) **
**HH**, n (%)	27 (7)	27 (100)	0 (0) **	24 (11)	3 (2) **
**ALF**, n (%)	208 (56)	24 (89)	184 (53) **	208 (100)	0 (0) **
**AKI**, n (%)	221 (59)	24 (89)	197 (57) **	135 (65)	86 (52) **
**Lowest platelet Count**, /mm^3^	133 [79–187]	60 [39–120]	137 [83–189] **	103 [56–168]	160 [113–215] *
**Lowest ScvO**_**2**_**/SvO**_**2**_, %	62 [56–66]	59 [54–65]	62 [57–66] *	61 [56–66]	63 [58–68] *
**BLOOD SAMPLE ON ADMISSION**					
**Lactate**, mEq l^-1^	5.1 [4.1–7.7]	8.2 [5.6–12.7]	4.9 [4.0–7.2] **	5.8 [4.4–9]	4.6 [3.9–6.0] **
**ScvO**_**2**_**/SvO**_**2**_, %	69 [64–74]	67 [60–74]	69 [64–74]	68 [63–74]	69 [64–74]
**AST**, IU/L	95 [47–193]	704 [160–1559]	86 [44–168] **	104 [51–235]	80 [41–164] *
**ALT**, IU/L	68 [32–153]	394 [135–782]	62 [31–128] **	69 [33–177]	64 [30–121] *
**LDH**, IU/L	336 [240–488]	918 [476–2325]	326 [235–449] *	356 [244–525]	322 [236–425] *
**AP**, IU/L	77 [58–106]	88 [68–153]	76 [58–104]	77 [58–114]	75 [59–102]
**GGT**, IU/L	68 [42–103]	77 [38–95]	68 [43–103]	66 [41–103]	71 [53–103]
**Total bilirubin**, mg dl^-1^	0.5 [0.3–0.9]	0.5 [0.4–1.1]	0.5 [0.3–0.9]	0.5 [0.3–0.8]	0.5 [0.3–0.9]
**APTT**, sec	32 [27–44]	41 [29–71]	32 [27–43]	38 [30–60]	29 [25–34] *
**PT**, %	65 [47–79]	46 [33–60]	67 [50–80] *	50 [38–69]	73 [64–88] *
** INR**	1.26 [1.12–1.54]	1.64 [1.35–2.18]	1.25 [1.11–1.49] *	1.22 [1.11–1.53]	1.30 [1.17–1.56]
**Platelets**, /mm^3^	201 [138–266]	169 [99–218]	202 [141–269]	169 [111–233]	220 [172–292] *
**Proteins**, mg dl^-1^	5.7 [5–6.3]	5.4 [4.7–5.9]	5.7 [5–6.4]	6.0 [5.0–6.0]	6.0 [5.0–7.0]
**Glucose**, mg dl^-1^	200 [155–289]	190 [143–237]	202 [158–291]	194 [147–294]	204 [163–282]
** pH**	7.28 [7.21–7.38]	7.22 [7.125–7.31]	7.30 [7.22–7.38] **	7.28 [7.17–7.38]	7.31 [7.23–7.38] *
**PaCO**_**2**_, mmHg	37 [33–44]	34 [29–41]	38 [33–44] *	37 [32–43]	38 [34–45]
**PaO**_**2**_, mmHg	111 [85–178]	113 [89–181]	111 [85–178]	119 [87–189]	106 [84–156]
**MAP**, mmHg	86 [75–103]	78 [70–91]	87 [76–105] **	83 [73–98]	94 [79–109] *
**Creatinine**, mg dl^-1^	1.2[0.9–1.6]	1.3 [1.1–2.25]	1.2 [0.9–1.6]	1.2 [1.0–2.0]	1.1 [0.9–1.4]
**CRP**, mg dl^-1^	40 [14–84]	45 [23–143]	37 [12–79]	44.5 [18.0–100.0]	32.5 [10–68.5] *

ICU = intensive care unit; CPR = cardiopulmonary resuscitation; ROSC = return of spontaneous circulation; TTM = targeted temperature management; COPD = chronic pulmonary obstructive disease; HIV = human immunodeficiency; IABP = intra-aortic balloon counterpulsation; ECMO = extracorporeal membrane oxygenation; CRRT = continuous renal replacement therapy; HH = hypoxic hepatitis; ALF = acute liver failure; AKI = acute liver failure; ScvO2/SvO2 = central venous oxygen saturation or mixed venous oxygen saturation; AST = aspartate aminotransferase; ALT = alanine aminotransferase; LDH = lactate dehydrogenase; AP = alkaline phosphatase; GGT = γ-glutamine transferase; APTT = activated partial thromboplastin time; PT = prothrombin time; INR = international normalized ratio; MAP = mean arterial pressure; CRP = C-reactive protein; APACHE = Acute Physiology and Chronic Health Evaluation; SOFA = Sequential Organ Failure Assessment.

* p < 0.05

** p < 0.01 for HH vs. no-HH or ALF vs. no-ALF.

### Acute liver failure and hypoxic hepatitis

ALF was observed in 208 patients (56%). The median time to ALF development was 3 [2–3] days. Patients with ALF had a longer time to return of spontaneous circulation (ROSC), were more likely to have had a non-shockable initial rhythm, received more adrenaline and more frequently received TTM than those who did not develop ALF; they were also more likely to receive vasopressor drugs, ECMO or CRRT during the ICU stay (Table 1). Patients with ALF received less frequently paracetamol (S1 Table), more often developed shock and HH, had higher blood lactate concentrations on admission and had lower lowest ScvO_2_/SvO_2_ values. Hospital mortality was significantly higher in patients with ALF than in those without (61% vs. 51%, p<0.05).

HH developed in 27 (7%) patients. Median AST and ALT on admission were higher in patients who developed HH than in those who did not, as were LDH and INR values (Table 1). The median time to HH development was 1 [0–1] days and the time to the highest AST/ALT values in these patients was 1 [0–1] days. Patients who developed HH had a longer time to ROSC, had more frequently had a non-shockable initial rhythm and received more adrenaline than those who did not develop HH; they were also more frequently treated with vasopressors, inotropic agents, ECMO and CRRT during the ICU stay (Table 1). Patients with HH received less frequently paracetamol, β-lactams or amiodarone (S1 Table) had higher lactate concentrations on admission, more frequently developed shock during the ICU stay, and had lower lowest ScvO_2_/SvO_2_ values. ALF occurred more frequently in patients who developed HH than in those who did not. ICU and hospital mortality rates and rates of unfavourable neurological outcome were significantly higher in patients with HH than in those without.

**Fig 1:**
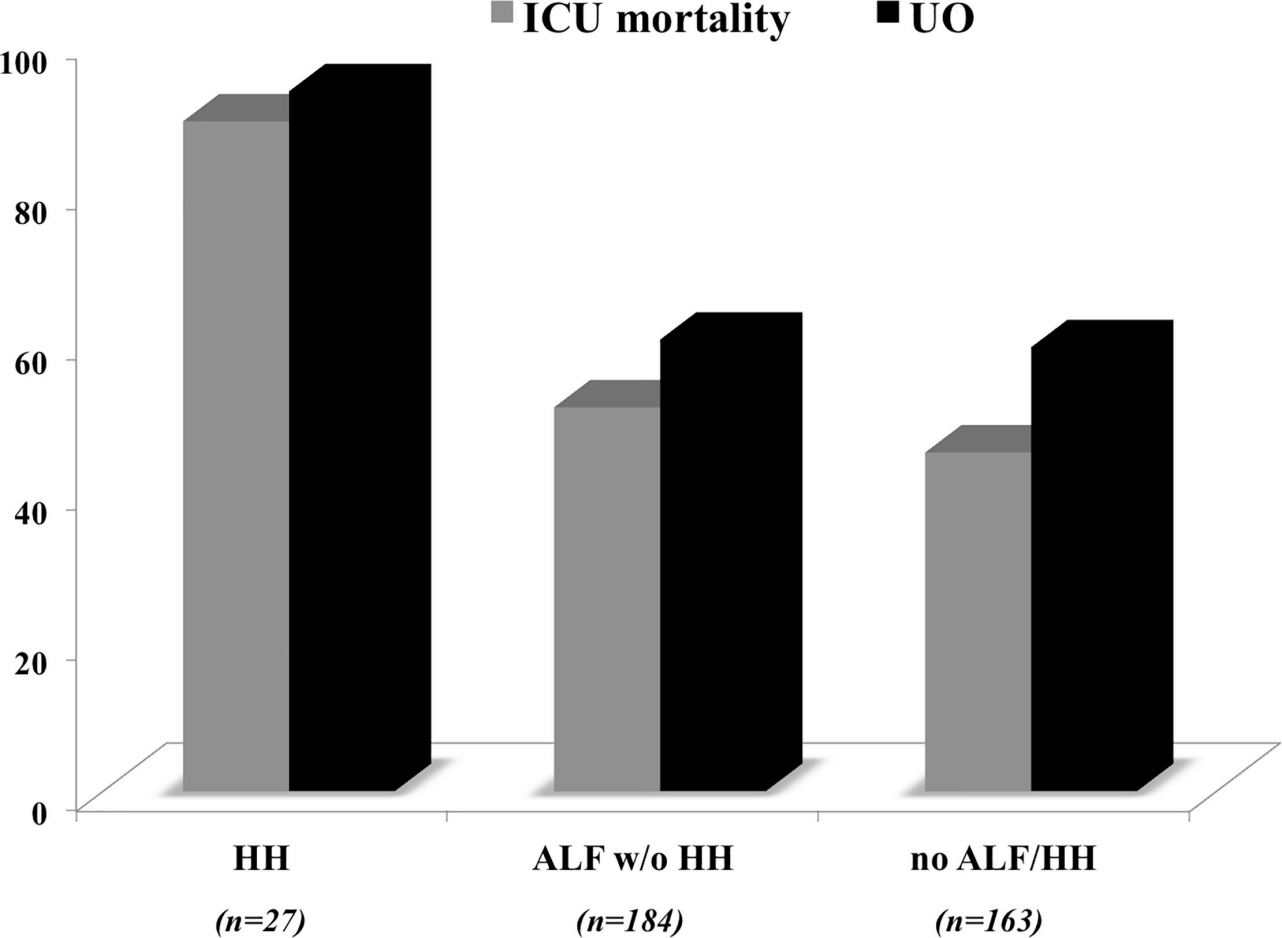
Intensive care unit (ICU) mortality and unfavourable neurological outcome (UO) according to the occurrence of hypoxic hepatitis (HH) or acute liver failure (ALF).

Patients who developed HH had higher mortality rates and rates of unfavourable neurological outcome compared to those with ALF without HH (n = 184) and to those without ALF or HH (n = 163; p = 0.03) (Fig 1).

### Neurological outcome

Compared to those with favourable neurological outcome, patients with an unfavourable neurological outcome were older and less likely to have had a witnessed arrest, to have received bystander CPR and to have had an initial shockable rhythm; time to ROSC was longer and adrenaline dose higher in patients with an unfavourable neurological outcome (Table 2). Patients with an unfavourable neurological outcome also had a higher lactate concentration and lower mean arterial pressure on admission and more frequently developed shock, and required vasopressors during the ICU stay than those with a favourable neurological outcome. HH occurred more frequently in patients with an unfavourable neurological outcome than in those with a favourable neurological outcome (25/226 vs. 2/148, p = 0.002).

**Table 2:** Characteristics of the study population according to hospital survival and neurological outcome.

	SURVIVORS (n = 161)			NON-SURVIVORS (n = 213)	Favourable neurological outcome (n = 148)	Unfavourable neurological outcome (n = 226)
**DEMOGRAPHICS**						
**Age**, years	57 [49–70]			66 [54–76] **	58 [51–70]	66 [52–76] **
**Weight**, Kgs	78 [70–85]			77 [65–85]	78 [70–85]	76 [65–85]
**Male**, n (%)	119 (74)			151 (71)	109 (74)	161 (71)
**ICU length of stay**, days	7 [4–13]			3 [2–7] **	6 [3–11]	3 [2–8] **
**APACHE II score**	23 [20–27]			26 [19–30] *	23 [20–27]	26 [20–30] *
**SOFA score**	10 [8–12]			13 [10–15] **	10 [8–12]	12 [10–14] **
**ARREST CHARACTERISTICS**						
**Witnessed**, n (%)	148 (92)			172 (81) **	136 (92)	184 (81) **
**Bystander CPR**, n (%)	121 (75)			133 (62) **	114 (77)	140 (62) **
**Time to ROSC**, min	12 [5–20]			17 [10–25] **	11.5 [5–20]	17 [10–25] **
**Adrenaline**, mg	2 [1–4]			4 [2–6] **	2 [1–4]	4 [2–6] **
**Out-of-hospital**, n (%)	91 (57)			116 (54)	82 (55)	125 (55)
**TTM**, n (%)	138 (86)			193 (91)	126 (85)	205 (91)
**Non-cardiac cause**, n (%)	54 (34)			99 (46) *	49 (33)	104 (46) *
**Non-shockable rhythm**, n (%)	67 (42)			154 (72) **	61 (41)	160 (71) **
**ICU mortality**, n (%)	0 (0)			194 (91) **	0 (0)	194 (86) **
**Hospital mortality**, n (%)	0 (0)			213 (100) **	0 (0)	213 (94) **
**Unfavourable neurological outcome at 3 months**, n (%)	13 (8)			213 (100) **	0 (0)	226 (100) **
**COMORBIDITIES**						
**Chronic heart failure**, n (%)	30 (19)			48 (23)	28 (19)	50 (22)
**Hypertension**, n (%)	73 (45)			86 (40)	65 (44)	94 (42)
**Coronary artery disease**, n (%)	59 (37)			87 (41)	55 (37)	91 (40)
**Diabetes,** n (%)	33 (20)			58 (27)	30 (20)	61 (27)
**COPD/Asthma**, n (%)	21 (13)			42 (20)	19 (13)	44 (19)
**Neurological disease**, n (%)	16 (10)			38 (18)	13 (9)	41 (18) *
**Chronic renal failure**, n (%)	23 (14)			39 (18)	21 (14)	41 (18)
**Liver cirrhosis**, n (%)	3 (2)			14 (7)	3 (2)	14 (6)
**HIV**, n (%)	2 (1)			1 (0)	2 (1)	1 (0)
**Corticosteroids**, n (%) **Chronic anticoagulation, n (%)**	27 (17) 27 (17)			58 (27) ** 38 (18)	25 (17) 25 (17)	60 (27) 40 (18)
**DURING ICU STAY**						
**Infection**, n (%)	110 (68)			131 (62)	98 (66)	143 (63)
**IABP**, n (%)	7 (4)			17 (8)	6 (4)	18 (8)
**ECMO**, n (%)	20 (12)			27 (13)	18 (12)	29 (13)
**Shock**, n (%)	67 (42)			133 (62) **	64 (43)	136 (60) **
**Vasopressor therapy**, n (%)	107 (66)			176 (83) **	100 (68)	183 (81) **
**Inotropic agents**, n (%)	80 (50)			121 (57)	73 (49)	128 (57)
**Mechanical ventilation**, n (%)	161 (100)			213 (100)	148 (100)	226 (100)
** At least one hepatotoxic drug, n (%)**	103 (64)			151 (71)	100 (68)	154 (68)
**CRRT**, n (%)	23 (14)			38 (18)	21 (14)	40 (18)
**HH**, n (%)	3 (2)			24 (11) **	2 (1)	25 (11) **
**ALF**, n (%)	80 (50)			128 (60)	75 (51)	13 (59)
**AKI**, n (%)	76 (47)			145 (68) **	70 (47)	151 (67) **
**Lowest platelet count**, /mm^3^	138 [94–181]			130 [70–188]	135 [94–180]	131 [70–190]
**Lowest ScvO**_**2**_**/SvO**_**2**_, %	61 [56–66]			63 [57–67]	61 [56–66]	62.65 [57–67]
**BLOOD SAMPLE ON ADMISSION**						
**Lactate**, mEq l^-1^	4.6 [3.8–6.2]			5.5 [4.2–8.5] **	4.6 [3.8–6.4]	5.3 [4.2–8.2] **
**ScvO**_**2**_**/SvO**_**2**_, %	68 [64–73]			69 [64–75]	67 [63–73]	69 [64–76]
**AST**, IU/L	84 [40–209]			99 [51–182]	82 [38–206]	102 [52–186]
**ALT**, IU/L	68 [30–158]			66 [32–141]	66.5 [29–155]	69 [33–144]
**LDH**, IU/L	308 [225–461]			352 [250–498]	296 [222–429]	360 [251–507]
**AP**, IU/L	69 [54–91]			84 [62–119]	68.5 [53–90]	83 [62–118]
**GGT**, IU/L	60 [37–91]			77 [45–110]	59 [36–91]	77 [45–107]
**Total bilirubin**, mg dl^-1^	0.5 [0.3–0.8]			0.6 [0.4–1]	0.5 [0.3–0.80]	0.5 [0.4–1]
**APTT**, sec	32 [27–42]			33 [28–45]	32 [27–45]	33 [28–46]
**PT**, %	69 [51–85]			62 [46–75]	69 [51–85]	62 [46–75]
** INR**	1.2 [1.1–1.5]			1.3 [1.2–1.6]	1.2 [1.1–1.5]	1.3 [1.2–1.6]
**Platelets**, /mm^3^	203 [147–261]			193 [127–268]	203 [142–263]	197 [131–267]
**Proteins**, mg dl^-1^	5.7 [5.1–6.3]			5.6 [5–6.3]	5.7 [5–6.2]	5.7 [5–6.4]
**Glucose**, mg dl^-1^	194 [147–274]			204 [160–293]	193 [145–276]	204 [166–293]
** pH**		7.31 [7.23–7.38]		7.29 [7.18–7.38]	7.3 [7.22–7.38]	7.29 [7.19–7.39]
**PaCO**_**2**_, mmHg		39 [33–44]		37 [32–43]	38 [33–44]	37 [32–43.75]
**PaO**_**2**_, mmHg		120 [87–175]		108 [84–182] *	120 [86–174]	109 [85–182] *
**MAP**, mmHg		93 [80–110]		83 [72–100] *	93 [80–110]	83.5 [73–100] *
**Creatinine**, mg dl^-1^		1.1 [0.9–1.5]		1.2 [0.9–1.7]	1.1 [0.9–1.5]	1.2 [0.9–1.7]
**CRP**, mg dl^-1^		31 [10–74]		44 [19–94] *	31 [10–72]	44 [18–92] *

ICU = intensive care unit; CPR = cardiopulmonary resuscitation; ROSC = return of spontaneous circulation; TTM = targeted temperature management; COPD = chronic pulmonary obstructive disease; HIV = human immunodeficiency; IABP = intra-aortic balloon counterpulsation; ECMO = extracorporeal membrane oxygenation; CRRT = continuous renal replacement therapy; HH = hypoxic hepatitis; ALF = acute liver failure; AKI = acute liver failure; ScvO2/SvO2 = central venous oxygen saturation or mixed venous oxygen saturation; AST = aspartate aminotransferase; ALT = alanine aminotransferase; LDH = lactate dehydrogenase; AP = alkaline phosphatase; GGT = γ-glutamine transferase; APTT = activated partial thromboplastin time; PT = prothtombin time; INR = international normalized ratio; MAP = mean arterial pressure; CRP = C-reactive protein; APACHE = Acute Physiology and Chronic Health Evaluation; SOFA = Sequential Organ Failure Assessment.

* p < 0.05

** p < 0.01 for survivors vs. non-survivors or favourable vs. unfavourable neurological outcome.

### Multivariable analyses

In multivariable logistic regression analysis, shock, chronic heart failure, high lactate concentration on admission, lowest venous (central or mixed) oxygen saturation, and use of CRRT during the ICU stay were independent predictors for development of ALF during the ICU stay (Table 3). High doses of adrenaline during CPR, high lactate concentration on admission and use of CRRT during the ICU stay were independent predictors for development of HH during the ICU stay (Table 3).

**Table 3:** Logistic regression analysis for predictors of acute liver failure and hypoxic hepatitis after cardiac arrest.

*Variable*	*OR*	*95% Confidence Interval*	*P value*
**ACUTE LIVER FAILURE**			
**Shock**	1.998	1.262 to 3.306	0.04
**Chronic heart failure**	1.858	1.044 to 3.540	0.049
**Lactate on admission**, mEq*L^-1^	1.224	1.120 to 1.339	<0.001
**Lowest ScvO**_**2**_**/SvO**_**2**_	0.972	0.946 to 0.999	0.04
**CRRT**	2.062	1.019 to 4.173	0.04
**HYPOXIC HEPATITIS**			
**Adrenaline**, mg	1.779	1.624 to 1.845	0.03
**Lactate on admission**, mEq*L^-1^	1.151	1.080 to 1.353	0.03
**CRRT**	1.279	1.849 to 51.137	0.008

OR = odds ratio; CRRT = continuous renal replacement therapy; ScvO_2_/SvO_2_, central/mixed venous oxygen saturation.

Unwitnessed arrest, non-shockable initial rhythm, lack of bystander CPR, high adrenaline doses and the development of AKI were all independent predictors of an unfavourable neurological outcome (Table 4); HH, but not ALF, was also a significant risk factor for poor neurological outcome.

**Table 4:** Logistic regression analysis for predictors of unfavourable neurological outcome.

Variable	OR	95% Confidence Interval	P value
**Witnessed CA**	0.892	0.755 to 0.911	0.02
**Bystander CPR**	0.867	0.712 to 0.955	0.03
**Non-shockable rhythm**	2.923	1.528 to 5.592	0.001
**Adrenaline**, mg	1.179	1.024 to 1.357	0.02
**AKI**	2.360	1.228 to 4.536	0.01
**HH**	16.276	2.625 to 81.345	0.003

OR = odds ratio; AKI = acute kidney injury; CA = cardiac arrest; CPR = cardiopulmonary resuscitation; HH = hypoxic hepatitis.

## Discussion

In our study, HH and ALF were observed in 7% and 56%, respectively, of patients resuscitated after CA, HH patients more frequently had an unfavourable neurological outcome compared to those with ALF without HH or those without ALF or HH. High dose adrenaline during CPR, high lactate concentration on admission and the need for CRRT were independent predictors of HH development. Development of HH, but not of ALF, was a significant risk factor for poor neurological outcome and ICU mortality.

Few studies have reported data on the development of liver dysfunction after CA; in one large database, liver failure, defined using only bilirubin levels, occurred in 10% of CA patients on ICU admission and in up to 20% during the ICU stay, with no differences between patients with favourable or unfavourable neurological outcome [18]. The occurrence of ALF in our study is quite high; however, some high bilirubin levels may have been due to hemolysis or rhabdomyolysis and increased INR levels could have been secondary to sepsis or previous anticoagulant therapy, which may have resulted in an overestimation of the number of cases. In two other studies, the incidence of HH ranged from 11% to 14% in patients with OHCA [8, 19]. CA is one of the main causes of HH in critically ill patients [20], and the prevalence of HH in heterogeneous critically ill populations is much lower, ranging from 1% to 12% [21, 22], depending on the threshold of AST/ALT used to define HH. Interestingly, we did not observe a higher incidence of ALF or HH in IHCA patients, in whom the presence of a pre-existing pathological condition might have reduced the tolerance of the liver to an ischemic injury, compared to OHCA patients.

The total dose of adrenaline during resuscitation, lactate concentrations on admission and use of CRRT were independent variables associated with the occurrence of HH. Conversely, Oh et al. reported that no-flow time was the only predictor of HH [19], and Champigneulle et al. demonstrated that the delay from collapse to ROSC was the major predictor of HH [8]. However, the retrospective design of all these studies makes it difficult to accurately assess the time to CA and initiation of CPR [23]. In our study, we included patients with IHCA, which may have added an additional confounder regarding the role of resuscitation times on the occurrence of liver failure. Nevertheless, in our population, high lactate concentration on admission and the total amount of adrenaline received during resuscitation, which can be considered as markers of the severity of the anoxic insult, better represented the extent of the ischemia/reperfusion injury on the liver than did the duration of resuscitation. In addition, experimental animal models of CA have reported that adrenaline may worsen the function of all organs, and in particular mesenteric perfusion, and its administration may lead to lower abdominal perfusion and higher lactate concentrations than other vasoconstrictor drugs [24, 25]. Finally, the use of CRRT could be considered as an index of patient severity or reflect the degree of the post-anoxic systemic inflammatory response, leading to MOF. Recently, Tujjar et al showed that 43% of patients resuscitated after CA developed AKI, and one third of these patients required CRRT; the total amount of adrenaline was independently associated with the development of AKI, because the adrenaline impaired abdominal perfusion [10].

The factors associated with development of ALF were shock, chronic heart failure, high lactate concentration on admission, low venous oxygen saturation and the use of CRRT. Although shock is not one of the most common causes of ALF [26], significant alterations in liver function are observed in up to 20% of patients with severe cardiogenic shock treated with veno-arterial ECMO [27]. Patients with chronic heart failure are at high-risk of developing cardiogenic shock, which requires aggressive therapy, including IABP and assist devices [28]. Low values of venous oxygen saturation, together with high lactate concentrations, may suggest the presence of a severe low cardiac output status that would result in reduced hepatic blood flow and oxygenation, as well as hepatic congestion from venous outflow obstruction, which would finally lead to liver failure [29].

The mortality rate in our patients with HH was 89%, which is consistent with rates reported by other authors (75–86%) [8, 19]. Our results showed that HH was also a significant predictor of poor neurologic outcome after CA, and previous studies have described an association of HH with mortality in the same setting [8, 19]. In two studies [5, 18], hepatic failure, assessed by the hepatic SOFA score, was not associated with mortality; however, this score does not consider AST/ALT as a relevant marker of hepatic damage induced by anoxia. Our findings showing a role of HH and AKI as predictors of unfavourable neurological outcome support the need to better describe the occurrence of extra-cerebral organ dysfunction among patients admitted to the ICU after CA. These abnormalities may influence patient outcome and need to be recognized early and further studies performed to identify potential therapies.

Our study has some strength. We underlined the need to assess the development of HH after both OHCA and IHCA, as this may represent a significant predictor of poor outcome. We have also shown some important predictors of HH in this patients’ population, i.e. the total dose of adrenaline during resuscitation, high lactate concentrations and the use of CRRT. The presence of these factors, together with a prolonged duration of resuscitation [8, 19], could identify those patients requiring a more accurate hemodynamic monitoring and/or the optimization of supporting therapies in order to minimize further liver injury. Also, we have included in our analysis the development of acute liver failure, which was not considered in previous studies [8, 19]. Finally, we collected a larger amount of data, including biological findings, therapeutic data and the use of potentially hepatotoxic drugs, which provided a more accurate analysis of the variables independently associated with the occurrence of post-anoxic liver injury.

Our study has also several limitations. First, we only collected routine measures of liver function and injury, and liver function may have been better assessed using arterial ammonium levels or functional tests (e.g., indocyanine green clearance test or 13C- methacetin breath test). Also, additional tests of the biosynthetic capacity of the liver, including blood-clotting factors or cholinesterases activity, which are reduced in patients with liver dysfunction, were not routinely performed. Second, we analyzed a mixed population of patients with OHCA and IHCA, who may have different predisposing conditions for the development of ALF and HH; however, the location of arrest was not predictive of the development of HH or of unfavourable neurological outcome in multivariable analysis. Third, due to the design of the study, we cannot determine whether there is a causal relationship between and the development of HH and unfavourable neurological outcome, which may just reflect the intensity of the initial anoxic injury. Fourth, we did not routinely perform morphologic examinations of the liver (e.g., ultrasound); however, hepatic echography, although of interest to exclude other causes of ischemic liver injury (vascular clotting) and to identify signs of chronic liver disease, may have limited diagnostic/prognostic role in the setting of HH.

## Conclusions

Hypoxic liver injury is rare after cardiac arrest but is an independent predictor of poor outcome. Data on the occurrence of extra-cerebral organ dysfunction should be collected in future studies dealing with prognostication and therapeutic interventions in this setting.

## Supporting information

S1 Table Presence of different hepatotoxic drugs, according to the occurrence of hypoxic hepatitis (HH) and acute liver failure (ALF) or according to patients’ outcome. (DOCX)
