# Supplementary material for: Acute liver dysfunction after cardiac arrest
Source: PLoS One. 2018 Nov 5;13(11):e0206655. doi: 10.1371/journal.pone.0206655 (PMC6218055; doi:10.1371/journal.pone.0206655)
Supplement: S1 Table — (DOCX) [file pone.0206655.s001.docx]

**Supplemental Table 1.** Presence of different hepatotoxic drugs, according to the occurrence of hypoxic hepatitis (HH) and acute liver failure (ALF) or according to patients’ outcome.

|  | **ALL**  **(n=374)** | **HH**  **(n=27)** | **No-HH**  **(n=347)** | **ALF**  **(n=208)** | **No-ALF**  **(n=166)** | **Survivors**  **(n=161)** | **Non-survivors**  **(n=213)** | **FO**  **(n=148)** | **UO**  **(n=226)** |
| --- | --- | --- | --- | --- | --- | --- | --- | --- | --- |
| **At least one hepatotoxic drug, n (%)** | 254 (68) | 10 (37) | 244 (70) * | 142 (68) | 112 (67) | 103 (64) | 151 (71) | 100 (68) | 154 (68) |
| **Paracetamol, n (%)** | 203 (54) | 7 (26) | 196 (56) * | 83 (40) | 120 (72) * | 101 (63) | 102 (48) * | 91 (62) | 112 (50) * |
| **Amiodarone, n (%)** | 187 (50) | 5 (19) | 182 (52) * | 100 (48) | 87 (52) | 79 (49) | 108 (51) | 73 (49) | 114 (50) |
| **β-lactams, n (%)** | 158 (42) | 4 (15) | 154 (44) * | 87 (42) | 71 (43) | 65 (40) | 93 (44) | 60 (41) | 98 (43) |
| **Quinolones, n (%)** | 7 (2) | 1 (4) | 6 (2) | 3 (1) | 4 (2) | 2 (1) | 5 (2) | 2 (1) | 5 (2) |
| **Azoles, n (%)** | 8 (2) | 2 (8) | 6 (2) | 2 (1) | 6 (4) | 1 (0.5) | 7 (3) | 1 (0.5) | 7 (3) |
| **Isoniazid, n (%)** | 0 (0) | 0 (0) | 0 (0) | 0 (0) | 0 (0) | 0 (0) | 0 (0) | 0 (0) | 0 (0) |
| **TMT/SMT, n (%)** | 0 (0) | 0 (0) | 0 (0) | 0 (0) | 0 (0) | 0 (0) | 0 (0) | 0 (0) | 0 (0) |
| **Metronidazole, n (%)** | 2 (0.1) | 0 (0) | 2 (0.1) | 1 (0.1) | 1 (0.1( | 0 (0) | 2 (0.1) | 0 (0) | 2 (0.1) |
| **Chemotherapy, n (%)** | 0 (0) | 0 (0) | 0 (0) | 0 (0) | 0 (0) | 0 (0) | 0 (0) | 0 (0) | 0 (0) |

* p <0.05 for HH vs. no-HH OR ALF vs. No-ALF OR survivors vs. non-survivors OR FO (favourable) vs. UO (unfavourable neurological outcome)

TMT/SMT = trimethoprim/ sulfamethoxazole
